# A Case of Cutaneous Tuberculosis in a Buruli Ulcer–Endemic Area

**DOI:** 10.1371/journal.pntd.0001751

**Published:** 2012-08-28

**Authors:** Martin W. Bratschi, Earnest Njih Tabah, Miriam Bolz, David Stucki, Sonia Borrell, Sebastien Gagneux, Blanbin Noumen-Djeunga, Thomas Junghanss, Alphonse Um Boock, Gerd Pluschke

**Affiliations:** 1 Swiss Tropical and Public Health Institute, Basel, Switzerland; 2 University of Basel, Basel, Switzerland; 3 National Committee for Leprosy, Buruli Ulcer, Yaws and Leishmaniasis Control, Department of Disease Control, Ministry of Public Health, Yaoundé, Cameroon; 4 Bankim District Hospital, Bankim, Cameroon; 5 Universität Heidelberg, Heidelberg, Germany; 6 FAIRMED Africa Regional Office, Yaoundé, Cameroon; University of Tennessee, United States of America

## Presentation of Case

**Figure 1:**
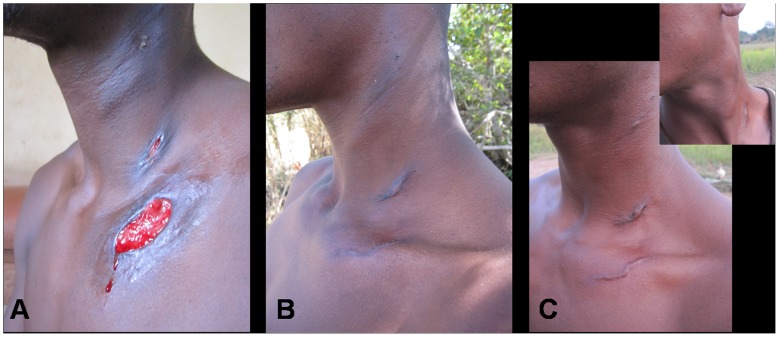
Clinical evolution of TB patient discovered in BU-endemic area. A 27-year-old male presented to an integrated health centre in a BU-endemic area in Cameroon with multiple lesions on the neck and upper chest. After acid-fast bacilli were observed in the wound exudates, the patient was diagnosed with BU and treated according to WHO guidelines. Figure 1A shows the patient on day 17 of BU treatment. The lesions healed following eight weeks of rifampicin/streptomycin combination therapy. Figure 1B and C show the patient 86 and 178 days after completion of BU treatment, respectively. Further laboratory analysis on the original wound exudates showed that the patient was suffering from TB as opposed to BU, and the appropriate long-term TB treatment was administered.

A 27-year-old male farmer presented himself to an integrated health centre in the Bankim health district of the Adamaoua region of Cameroon with two ulcerative lesions with undermined edges on the upper chest and neck as well as enlarged and indurated lymph nodes of the neck (see Figure 1). He had not sought any traditional treatment and indicated that the condition had been ongoing for one month. Given the known high prevalence of *Mycobacterium ulcerans (M. ulcerans)* disease (Buruli ulcer) in the Bankim health district [1], ulcer exudates were examined for acid-fast bacilli by Ziehl-Neelsen (ZN) staining at the local health centre and tested positive. Based on the positive ZN stain, a diagnosis of Buruli ulcer (BU) was made and the treatment recommended for BU by the World Health Organization (WHO) [2], daily rifampicin (600 mg *p.o.*) and streptomycin (1 g *i.m.*) for 56 days, was administered to the patient. Additional swabs of ulcer exudates were analyzed using the *M. ulcerans*–specific IS2404 quantitative polymerase chain reaction (qPCR) assay [3]. All four swabs obtained from the patient tested negative. Exudate swabs were also used for the initiation of a culture on Löwensein-Jensen medium after decontamination with 2.5% oxalic acid for 30 minutes at room temperature. After 8.5 weeks of incubation at 30°C, the optimal growth temperature of *M. ulcerans*, mycobacterial growth was observed. The cultured mycobacteria were ZN positive, but negative in the *M. ulcerans*–specific IS2404 qPCR. PCR amplification [4] and DNA sequencing of the rifampicin resistance determining region (RRDR) of the *rpoB* gene identified the strain as belonging to the *M. tuberculosis* complex; no rifampicin resistance conferring mutation in the RRDR was found. A qPCR identifying a single-nucleotide (A to C) change at position 2′154′724 further characterized the cultivated strain as belonging to Lineage 4 (Euro-American Linage) of *M. tuberculosis* [5]. Spoligotyping and analysis using the SITVITWEB database revealed that the strain belonged to the “T-family” [6] of *M. tuberculosis*, a spoligotype of Lineage 4 which encompasses all strains that are difficult to classify into other spoligotype families. While the strain therefore does not belong to the “Cameroon Family” of TB, the obtained spoligo type has previously been reported to occur in Cameroon [6].

Based on this laboratory diagnosis of an *M. tuberculosis* infection, the patient was re-examined 186 days after completion of the BU treatment. At this point, the ulcers had fully scarred (see Figure 1). However, the lymph nodes of the neck remained enlarged and indurated. A chest X-ray provided no evidence for pulmonary tuberculosis (TB), and the patient tested negative for human immunodeficiency virus (HIV) infection. Given the laboratory results and the clinical presentation, the patient was retrospectively diagnosed as a case of cutaneous TB [7]. Given the insufficiency of the BU treatment to cure TB, the patient was started on the full regimen of the standard TB treatment recommended by the Cameroon National TB Control Program: two months of isoniazid, rifampicin, ethambutol, and pyrazinamide followed by four months of isoniazid and rifampicin.

## Case Discussion

Ethical approval (clearance N° 041/CNE/DNM/09, 19/06/2009) to analyze patient specimens was obtained from the National Ethics Committee of Cameroon, registered under the N° IRB00001954. Written informed consent from the patient was obtained before specimens were used for reconfirmation of clinical diagnosis and detailed laboratory analysis.

BU disease presents with a variety of clinical manifestations including nonulcerative forms such as movable subcutaneous nodules, plaques, and oedema, which may eventually progress to ulcerative lesions with characteristic undermined edges. Without treatment, ulcers may enlarge considerably and involve entire limbs or large areas of the trunk [8]. It is believed that mycolactone, the macrolide toxin produced by *M. ulcerans*, largely contributes to the pathogenesis of BU disease [9]. The diversity in clinical presentation renders clinical diagnosis difficult. Of the four currently available methods for laboratory reconfirmation of BU [8], only one, ZN microscopy, is suitable as a point-of-care diagnostic test in the African endemic areas, which are usually remote and rural. However, its sensitivity is limited [10], [11]. Cultivation of the slow-growing mycobacteria, histopathology, and PCR-based detection of *M. ulcerans* DNA can only be performed in central reference laboratories.

Given its wide range of clinical presentations, the differential diagnosis of cutaneous tuberculosis, which makes up 1%–2% of all TB cases worldwide [7], [12], is also difficult. Both infectious and noninfectious diseases of the skin need to be considered when examining a potential case of cutaneous TB [13]. For a definite diagnosis, histological examination, PCR, or optimally isolation of *M. tuberculosis* is required. All of these diagnostic methods again require sophisticated reference laboratories [7], [12]. Once diagnosed, patients can be treated with the regiment that is also used to treat pulmonary TB [7]. It is remarkable that a clinical manifestation very similar to that of BU disease was observed in the case presented here, although *M. tuberculosis* does not produce a potent macrolide toxin.

For patients from BU-endemic regions, ZN microscopy is usually accepted as reconfirmation of the clinical diagnosis and, to reduce costs, it has been suggested that only ZN negative swabs should be sent to a reference laboratory for analysis by PCR [11]. While the vast majority of ZN positive samples are also PCR positive, sensitivity of PCR is much higher than microscopy and many ZN negative samples still turn out PCR positive [11]. In BU-endemic areas as remote as the Bankim health district, clinical diagnosis by local medical staff is often considered sufficient for treatment decision. It has been shown that if clinical diagnosis is performed by highly trained and experienced staff, more than 90% of suspected cases can be reconfirmed by PCR [14]. However in regions where health care staff does not regularly encounter BU cases, a large proportion of suspected BU cases cannot be confirmed by laboratory tests [15]. Clinical overdiagnosis on one side, and true BU cases missed by relying only on ZN microscopy on the other side, can lead to over- or undertreatment of patients, respectively. It is therefore recommended that all BU cases should be laboratory confirmed [15]. If the decision is made to send only ZN negative swabs for confirmation to a reference laboratory, training of health care staff in BU differential diagnosis becomes even more important. Furthermore, such training should include the clinical presentation of other skin diseases, including other mycobacterial diseases. As demonstrated by the case presented here, lymphadenopathy—not a typical sign of BU—should lead to a more detailed clinical examination.

Overall, the case of a misdiagnosed patient with cutaneous TB in a BU-endemic area presented here further underscores the need for a simple, highly sensitive, and specific point-of-care diagnostic test for BU.

**Learning Points**

- Clinical diagnosis of Buruli ulcer should be supplemented with laboratory examinations.
- Microscopy, the only point-of-care laboratory test currently available, does not differentiate between Buruli ulcer and infections by other mycobacteria.
- Clinical presentation of other mycobacterial diseases should be included when training staff on the differential diagnosis of Buruli ulcer.
- There is a pressing need for a sensitive and specific point-of-care laboratory test for Buruli ulcer.
